# Case Report: Stevens-Johnson Syndrome and Hepatotoxicity Induced by Osimertinib Sequential to Pembrolizumab in a Patient With EGFR-Mutated Lung Adenocarcinoma

**DOI:** 10.3389/fphar.2021.672233

**Published:** 2021-08-12

**Authors:** Caterina Gianni, Giuseppe Bronte, Angelo Delmonte, Marco Angelo Burgio, Kalliopi Andrikou, Manlio Monti, Cecilia Menna, Giovanni Luca Frassineti, Lucio Crinò

**Affiliations:** Department of Medical Oncology, IRCCS Istituto Romagnolo per lo Studio dei Tumori (IRST) “Dino Amadori”, Meldola, Italy

**Keywords:** non-small cell lung cancer, pembrolizumab, osimertinib, stevens johnson syndrome, liver toxicity

## Abstract

**Background:** Lung cancer is a complex disease with many subtypes. However, histochemical characteristics, and genetic mutation determinations are contributing to better define therapeutic targets and new drugs. Although this guarantees patients the possibility of obtaining tailored treatment, it makes it more difficult for clinicians patient management more difficult for clinicians who have to define the most suitable therapeutic strategy and to deal with new treatment-related adverse events (TRAEs). It has been seen that the administration of a tyrosine kinase inhibitor (TKI) sequential to an immune checkpoint inhibitor (ICI) can lead to a higher rate of severe and life-threatening TRAEs. We report the case of a patient with advanced non-small cell lung cancer (NSCLC) who experienced severe hepatotoxicity and Stevens-Johnson syndrome (SJS) induced by osimertinib sequential to pembrolizumab.

**Case presentation:** A 54-year-old woman with advanced NSCLC received one cycle of chemotherapy plus pembrolizumab after diagnosis. Ten days later she began osimertinib 80 mg daily because epidermal growth factor receptor (EGFR) analysis had revealed an exon 19 deletion. On day 23 of osimertinib the patient experienced an episode of grade (G) 3 hepatotoxicity resolved by discontinuing osimertinib and corticosteroid therapy. The patient restarted osimertinib 80 mg daily after the remission of symptoms but was hospitalized 14 days later following a second episode of severe G3 hepatotoxicity and the onset of SJS, successfully treated with high-dose corticosteroids. Despite the short exposure to osimertinib, the patient obtained a good pathological response.

**Conclusion:** It is important to alert clinicians to carefully evaluate the sequential therapeutic strategy in patients with NSCLC who are candidates for TKI- or ICI-based treatment. Our experience suggests that the use of tyrosine kinase inhibitors (TKIs) as front-line treatment is a more reasonable and safe option for EGFR-mutated lung adenocarcinoma, with ICIs considered as a possible further treatment in sequential approaches.

## Introduction

Lung cancer is the leading cause of cancer death worldwide (Torre et al., 2016), with the majority of patients diagnosed with advanced non-small cell lung cancer (NSCLC). The prognosis for these patients is poor, with an estimated 5-year overall survival (OS) of around 15%. Recently, the identification of a number of molecular alterations has helped to identify oncogene-addicted tumors (Rosell and Karachaliou, 2016). The greatest benefit in OS has been achieved in patients harboring activating mutations in epidermal growth factor receptor (EGFR) gene or genetic rearrangements of echinoderm microtubule-associated protein-like 4 with anaplastic lymphoma kinase (EML4-ALK). Osimertinib targets both EGFR-activating and T790M mutations. A tumor response rate of 60% was reported in two single-arm trials, the phase I AURA (Jänne et al., 2015) and the phase II AURA2 (Goss et al., 2016) trials. In November 2015, the drug was approved by the Food and Drug Administration (FDA) for the treatment of patients with metastatic NSCLC and EGFR T790M mutation progressing during EGFR TKI treatment. The phase III AURA3 study (Mok et al., 2017) reported significantly longer progression-free survival (PFS) and a higher objective response rate (ORR) for osimertinib with respect to platinum plus pemetrexed combination chemotherapy in this population of patients. Hence, osimertinib was approved by the FDA and the European Medicines Agency (EMA) for use in NSCLC with sensitizing EGFR mutations (National Comprehensive Ca, 2021). Osimertinib is more tolerable than chemotherapy and can penetrate the blood brain barrier (Colclough et al., 2021).

The main treatment for patients with metastatic NSCLC and no evidence of oncogene drivers is a combination of an immune checkpoint inhibitor (ICI) with platinum-based chemotherapy (Gandhi et al., 2018). The combined use of pembrolizumab with pemetrexed and cisplatin or carboplatin has already been approved for clinical practice in patients with non-oncogene-addicted advanced NSCLC. A recent meta-analysis on chemoimmunotherapy highlighted that it does not induce a higher risk of high-grade hematological or gastrointestinal adverse effects, with the exception of high-grade diarrhea (Abdel-Rahman, 2019).

The use of these new biological treatments has also meant that oncologists have had to deal with rare new toxicities other than those deriving from chemotherapy. never experienced before. Of note, the sequential use of ICIs (especially PD-1 inhibitors) followed by TKIs for EGFR-mutated adenocarcinoma has been shown to increase the risk of severe and life-threatening side-effects, toxicities as seen in clinical trials with combination regimens (Schoenfeld et al., 2019; De-Rui Huang and Chih-Hsin Yang, 2020). Hence, a pressing challenge now facing clinicians is that one of the new challenges consists of being able to combine these new therapies in the right way to maximize therapeutic success and limit potentially dangerous adverse events.

## Case Presentation

A 54-year-old woman with NSCLC (ALK-negative, ROS 1-negative, PDL1 expression <1%, EGFR not available) without comorbidities presented with disseminated disease involving lymph nodes, adrenal glands, bones, and brain. She immediately began carboplatin 5AUC, pemetrexed 500 mg/m^2^ and pembrolizumab 200 mg q21 because of rapid disease spread. After the first therapy cycle, EGFR status was evaluated and showed an exon 19 deletion. Ten days later the patient started osimertinib 80 mg daily. On day 23 of osimertinib she interrupted treatment because of fever, grade (G) 3 hypertransaminasemia (Common Terminology Criter, 2020), with negative abdomen ultrasonography, suspected pancreatitis, and concomitant G2 mucositis. Upon suspicion of drug-induced hepatotoxicity, the patient began steroid therapy, slowly tapering off it. After an 8-day discontinuation the patient was re-started on osimertinib 8 mg/die because of the complete remission of symptoms and the improvement in liver serum enzymes to G1 (AstraZeneca Pharmaceuticals, 2018).

**FIGURE 1:**
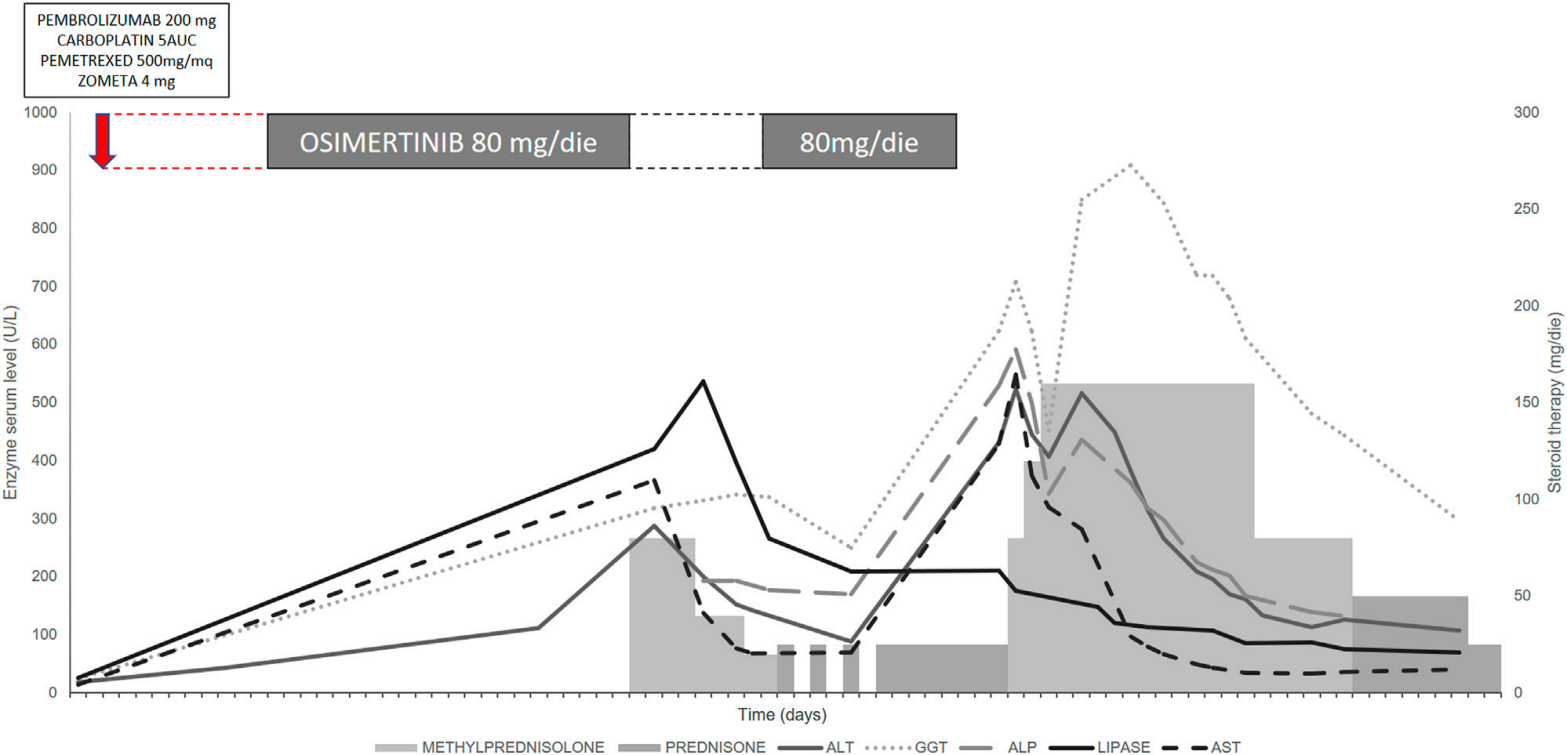
Serum enzyme trend during treatment with osimertinib after chemotherapy + pembrolizumab and concomitant steroid therapy. The modification of liver enzymes indicated grade (G) 3 liver injury, with important alterations in alkaline phosphatase (ALP), gamma-glutamyltransferase (GGT), aspartate transaminase (AST) and alanine transaminase (ALT). Although the interruption of osimertinib and the concomitant use of steroids reduced the altered enzyme values, an insufficient interval between the adverse event and osimertinib rechallenge rapidly induced new toxicity that took several days to resolve.

**FIGURE 2:**
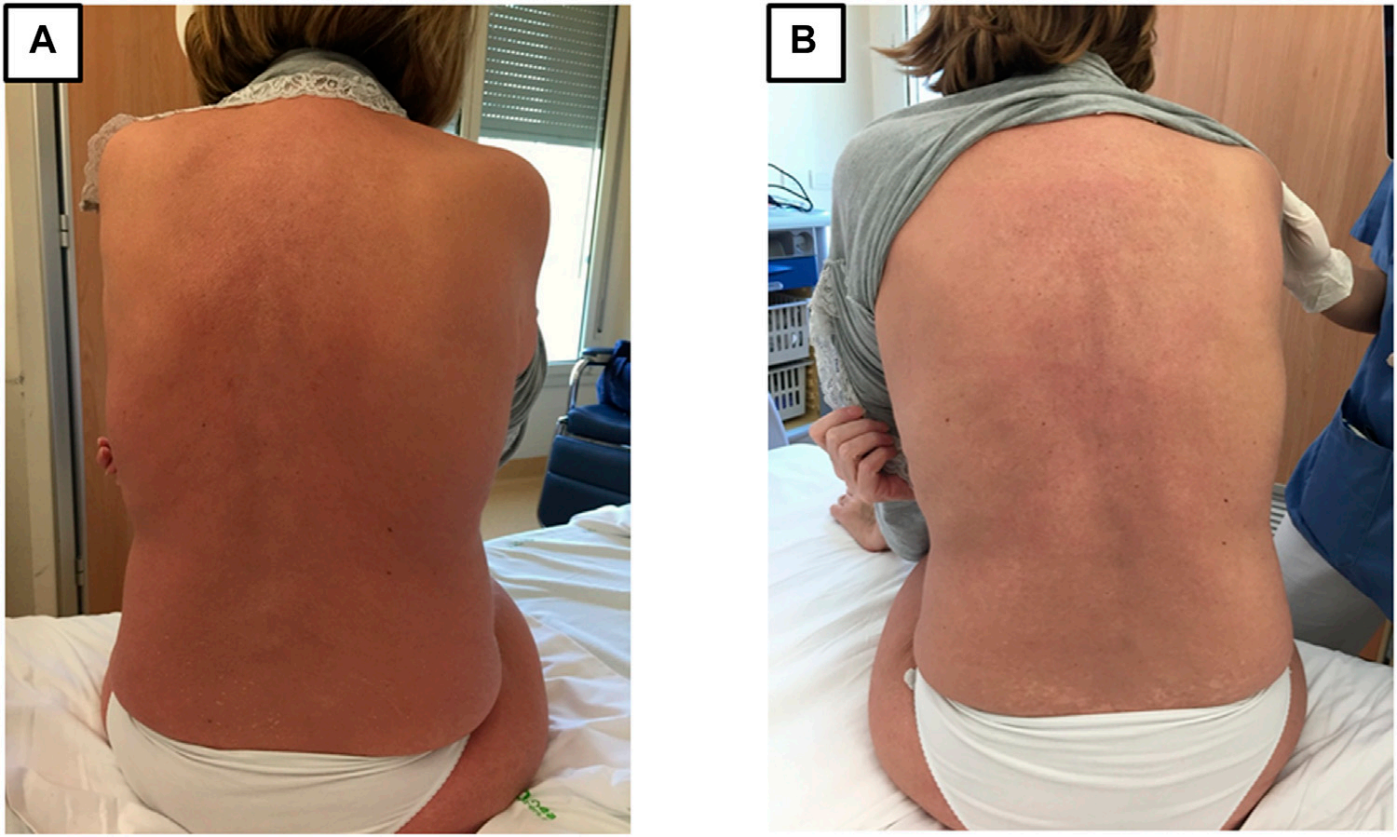
Cutaneous and mucosal involvement in Stevens-Johnson syndrome (day 45 of osimertinib). **(A)** Diffuse painful G3 erythema over the entire body upon hospital admission. The patient also had mucositis of the oral cavity (with blisters), pharynx (causing dysphagia), eyes, vagina, and nose (with ulcers causing episodes of epistaxis). SCORTEN score 3 (Bastuji-Garin, et al., 2000). **(B)** Reduction in the diffuse erythema, with areas of flaking and depigmentation (day 60).

Fourteen days after the rechallenge with osimertinib, the patient was hospitalized in a state of hypovolemic shock with fever >39°C, diffuse painful G3 cutaneous erythema with confluent macules, pruritus and diffuse flaking. Laboratory tests showed hepatotoxicity with a G3 increase in liver serum enzymes (Figure 1). Viral hepatitis was excluded and blood cultures were negative. After 24 h, the cutaneous toxicity worsened and was accompanied by diffuse mucositis with oral blisters, nasal ulcers and conjunctivitis. Nikolsky’s sign was negative. Finally, a clinical diagnosis of Stevens-Johnson syndrome was made (Figure 2).

Methylprednisolone (1 mg/kg/die) in association with an antihistamine were started, with rapid defervescence, and then increased to 2 mg/kg/die when the skin conditions got worse. An antimycotic (fluconazole) and empirical antibiotic therapies (piperacillin-tazobactam) were started intravenously, as were hyaluronate ocular drops, sucralfate oral granules, a ceramide-containing topical body cream and parenteral nutrition. Liver enzymes showed a slow downtrend and the cutaneous erythema began to resolve with, however, residual areas of depigmentation due to cutaneous flaking.

**FIGURE 3:**
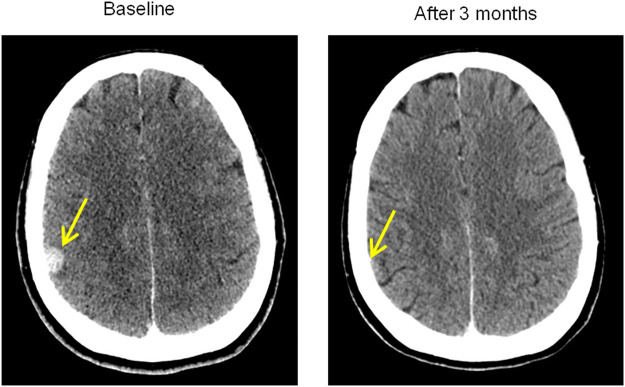
Brain response at CT scan. Comparison between baseline brain lesion at diagnosis and the impressive complete response after 3 months following chemoimmunotherapy and osimertinib. Osimertinib was only taken for a total of 37 days and then suspended because of severe toxicity.

A total body contrast-enhanced CT scan showed disease stability and complete response of brain lesions (day 60) (Figure 3). The patient was discharged and continued the tapering off of oral steroid therapy. The skin returned to normal, with residual areas of depigmentation that showed further cutaneous flaking, and liver enzymes slowly reached normal values. The patient was monitored closely for the next 3 months.

A subsequent positron emission tomography (PET) scan showed a slight increase in disease progression. In agreement with the patient, after receiving patient informed consent, osimertinib was resumed at a dose of 80 mg daily combined with prednisone 25 mg, with close clinical surveillance. The patient successfully continued osimertinib, slowly reducing corticosteroid therapy, with good tolerance and maintaining disease stability.

## Discussion

In our case report, we present an unusual combination of rare and life-threating adverse events. Although a few postmarketing cases of SJS have been reported in patients receiving osimertinib (AstraZeneca Pharmaceutica, 2018), elevation in liver enzymes is considered uncommon (5% G1-2, <1% G3) (Goss et al., 2016). In particular, osimertinib-induced acute liver injury is an extremely rare event and described in few case reports (Yoshida and Kim, 2017; Hirabayashi et al., 2018; González and Chatterjee, 2019). Conversely, immune-related hepatotoxicity has been documented as a more common ICI- mediated adverse event, as have as well for cutaneous reactions. SJS is a rare event that are described too (Schoenfeld et al., 2019; National Comprehensive Ca, 2021). There are several therapeutic options for lung cancer are various to date and finding the right treatment sequence is critical given that the association between ICIs and TKIs may be lethal (De-Rui Huang and Chih-Hsin Yang, 2020). In a recent study, the sequential use of ICIs (especially PD-1 inhibitors) and osimertinib appeared to increase the risk of toxicity, including pneumonitis and colitis (Sharp and Corp, 2021). Toxicity occurred irrespective of the duration of the PD-1 blockade treatment, usually a few weeks after beginning osimertinib (as in the case of our patient). The half-life of osimertinib is 55 h (Jänne et al., 2015), whereas the receptor occupancy of anti PD-1 antibodies can last for months and may also vary among patients (Brahmer et al., 2010). This may explain the long-term effectiveness of ICIs on disease response and thus, given this durable action, severe TREAs may also occur after several months of latency.

The mechanism behind the synergism between ICIs and TKI that is responsible for higher toxicity is still not clearly understood. As seen from histological findings in conditions of immune-related adverse events (iRAEs), pembrolizumab induces a modification of the immune activity in the tissue microenvironment, with an increase in infiltrating T-lymphocytes CD3^+^, especially CD8^+^ (Saw et al., 2017; Zen and Yeh, 2018). This may create a favorable environment for a cytotoxic reaction when osimertinib is administered. It can be hypothesized that osimertinib may have undefined immune effects capable of triggering an immune response in conditions of susceptibility induced by ICIs.

Although the association of TKIs and ICIs may have a therapeutic rationale (Karachaliou et al., 2017; Latif and Liu, 2019) given the potential impact of EGFR in immune signaling and induction of PD-L1 expression, no clear benefit have been reported for their sequential or combined use. Starting immunotherapy +/− chemotherapy as front-line treatment could prove a good choice when molecular results are not rapidly available, with the intention of switching to targeted therapy as soon as possible. However, the first administration of immunotherapy may compromise the further possibility of treatment with osimertinib or other TKIs. This is important because ICIs are currently used in various settings such as locally advanced unresectable stage III NSCLC, regardless of EGFR status, and as adjuvant/neoadjuvant treatment (Remon et al., 2020).

A safe wash-out period of at least 12 months between the last infusion of ICIs and the start of osimertinib should be considered to reduce the risk of severe toxicity (Brahmer et al., 2010) when this treatment sequence is needed. As PD-L1 has long-lasting receptor occupancy, its evaluation before starting TKIs could be useful to identify the minimum wash-out latency time after ICIs. When osimertinib is the best option for the patient, a desensitization method could be taken into account (Yoshida and Kim, 2017), but this attempt could also be dangerous when ICI therapy has already been administered.

Hypothesizing the involvement of an immune-mediated mechanism in the genesis of the toxicity, we successfully treated the adverse events of our patient with corticosteroid therapy, enabling her to continue treatment assuming an immune-mediated mechanism involved in the genesis of the toxicity (Coleman and Pontefract, 2016). One of the limits of our approach was that invasive investigations such as skin or liver biopsies were not carried out, as these would have been useful for a complete analysis of the case. A sequential approach with TKIs as a first treatment option appears to be the safest strategy in EGFR-mutant metastatic NSCLC to avoid severe immune.-mediated TRAEs, with ICIs reserved as possible further treatment.

## Conclusion

We reported a case of SJS and G3 hepatotoxicity induced by osimertinib administered sequentially to pembrolizumab plus chemotherapy and successfully treated with high-dose steroid therapy. There are still a great many uncertainties about the correct sequencing timing of sequence of ICIs and EGFR TKIs, and also about the correct management of potential toxicities. The identification of a patient population with fewer toxicity risks and safer TKIs should be considered is needed to reduce the risk of toxicity from sequential approaches. Further research is thus warranted into toxicity pathogenesis and safe treatment associations to maximize therapeutic success.

## Data Availability

The original contributions presented in the study are included in the article/Supplementary Material, further inquiries can be directed to the corresponding author.

## References

[B1] Abdel-Rahman O. (2019). Toxicity Patterns Associated with Chemotherapy/immune Checkpoint Inhibitor Combinations: a Meta-Analysis. Immunotherapy 11 (6), 543–554. 10.2217/imt-2018-0186 31135244

[B2] AstraZeneca Pharmaceuticals (2018). Tagrisso (Osimertinib) [package Insert]. U.S. Food and Drug Administration website. Available at: https://www.accessdata.fda.gov/drugsatfda_docs/label/2018/208065s008lbl.pdf (Revised April 2018, Accessed March 03 2020).

[B3] Bastuji-Garin S. Fouchard N. Bertocchi M. Roujeau J. C. Revuz J. Wolkenstein P. (2000). SCORTEN: a Severity-Of-Illness Score for Toxic Epidermal Necrolysis. J. Invest. Dermatol. 115 (2), 149–153. 10.1046/j.1523-1747.2000.00061.x 10951229

[B4] Brahmer J. R. Drake C. G. Wollner I. Powderly J. D. Picus J. Sharfman W. H. (2010). Phase I Study of Single-Agent Anti-programmed Death-1 (MDX-1106) in Refractory Solid Tumors: Safety, Clinical Activity, Pharmacodynamics, and Immunologic Correlates. J. Clin. Oncol. 28 (19), 3167–3175. 10.1200/JCO.2009.26.7609 20516446 PMC4834717

[B5] Colclough N. Chen K. Johnström P. Strittmatter N. Yan Y. Wrigley G. L. (2021). Preclinical Comparison of the Blood-Brain Barrier Permeability of Osimertinib with Other EGFR TKIs. Clin. Cancer Res. 27 (1), 189–201. 10.1158/1078-0432.CCR-19-1871 33028591

[B6] Coleman J. J. Pontefract S. K. (2016). Adverse Drug Reactions. Clin. Med. (Lond) 16 (5), 481–485. 10.7861/clinmedicine.16-5-481 27697815 PMC6297296

[B7] Common Terminology Criteria for Adverse Events (CTCAE) | Protocol Development | CTEP. Available at: https://ctep.cancer.gov/protocolDevelopment/electronic_applications/ctc.htm. (Accessed April 15, 2020).

[B8] De-Rui Huang D. Chih-Hsin Yang J. (2020). Checkpoint Inhibitor Combined with Tyrosine Kinase Inhibitor-The End or Beginning? J. Thorac. Oncol. 15 (3), 305–307. 10.1016/j.jtho.2019.12.121 32093849

[B9] Gandhi L. Rodríguez-Abreu D. Gadgeel S. Esteban E. Felip E. De Angelis F. (2018). Pembrolizumab Plus Chemotherapy in Metastatic Non-Small Cell Lung Cancer. N. Engl. J. Med. 378, 2078–2092. 10.1056/NEJMoa1801005 29658856

[B10] González I. Chatterjee D. (2019). Histopathological Features of Drug-Induced Liver Injury Secondary to Osimertinib. ACG Case Rep. J. 6 (2), e00011. 10.14309/crj.0000000000000011 31616716 PMC6657992

[B11] Goss G. Tsai C. M. Shepherd F. A. Bazhenova L. Lee J. S. Chang G. C. (2016). Osimertinib for Pretreated EGFR Thr790Met-Positive Advanced Non-Small Cell Lung Cancer (AURA2): a Multicentre, Open-Label, Single-Arm, Phase 2 Study. Lancet Oncol. 17 (12), 1643–1652. 10.1016/S1470-2045(16)30508-3 27751847

[B12] Hirabayashi R. Fujimoto D. Satsuma Y. Hirabatake M. Tomii K. (2018). Successful Oral Desensitization with Osimertinib Following Osimertinib-Induced Fever and Hepatotoxicity: A Case Report. Invest. New Drugs 36 (5), 952–954. 10.1007/s10637-018-0608-7 29721756

[B13] Jänne P. A. Yang J. C. Kim D. W. Planchard D. Ohe Y. Ramalingam S. S. (2015). AZD9291 in EGFR Inhibitor-Resistant Non-Small Cell Lung Cancer. N. Engl. J. Med. 372 (18), 1689–1699. 10.1056/NEJMoa1411817 25923549

[B14] Karachaliou N. Gonzalez-Cao M. Sosa A. Berenguer J. Bracht J. W. P. Ito M. (2017). The Combination of Checkpoint Immunotherapy and Targeted Therapy in Cancer. Ann. Transl Med. 5 (19), 1–10. 10.21037/atm.2017.06.47 29114546 PMC5653508

[B15] Latif H. Liu S. V. (2019). Combining Immunotherapy and Epidermal Growth Factor Receptor Kinase Inhibitors: worth the Risk? Ann. Transl Med. 7 (S3), S76. 10.21037/atm.2019.03.6 31576285 PMC6685870

[B16] Mok T. S. Wu Y-L. Ahn M-J. Garassino M. C. Kim H. R. Ramalingam S. S. (2017). Osimertinib or Platinum-Pemetrexed in EGFR T790M-Positive Lung Cancer. N. Engl. J. Med. 376 (7), 629–640. 10.1056/NEJMoa1612674 27959700 PMC6762027

[B17] National Comprehensive Cancer Network (2021). Non-Small Cell Lung Cancer Guidelines. Version 5. Available at: https://www.nccn.org/professionals/physician_gls/pdf/nscl.pdf (Accessed March 03, 2021).

[B18] Remon J. Passiglia F. Ahn M. J. Barlesi F. Forde P. M. Garon E. B. (2020). Immune Checkpoint Inhibitors in Thoracic Malignancies: Review of the Existing Evidence by an IASLC Expert Panel and Recommendations. J. Thorac. Oncol. 15 (20), 914–947. 10.1016/j.jtho.2020.03.006 32179179

[B19] Rosell R. Karachaliou N. (2016). Large-scale Screening for Somatic Mutations in Lung Cancer. Lancet 387 (10026), 1354–1356. 10.1016/S0140-6736(15)01125-3 26777918

[B20] Saw S. Lee H. Y. Ng Q. S. (2017). Pembrolizumab-induced Stevens-Johnson Syndrome in Non-melanoma Patients. Eur. J. Cancer 81, 237–239. 10.1016/j.ejca.2017.03.026 28438440

[B21] Schoenfeld A. J. Arbour K. C. Rizvi H. Iqbal A. N. Gadgeel S. M. Girshman J. (2019). Severe Immune-Related Adverse Events Are Common with Sequential PD-(L)1 Blockade and Osimertinib. Ann. Oncol. 30 (5), 839–844. 10.1093/annonc/mdz077 30847464 PMC7360149

[B22] Sharp M. Corp D. (2021). Keytruda (Pembrolizumab) [package Insert]. U.S. Food and Drug Administration website. Available at: https://www.accessdata.fda.gov/drugsatfda_docs/label/2021/125514s096lbl.pdf (Accessed March 2021, July 20 2021).

[B23] Torre L. A. Siegel R. L. Ward E. M. Jemal A. (2016). Global Cancer Incidence and Mortality Rates and Trends--An Update. Cancer Epidemiol. Biomarkers Prev. 25 (1), 16–27. 10.1158/1055-9965.EPI-15-0578 26667886

[B24] Yoshida H. Kim Y. H. (2017). Successful Osimertinib Rechallenge after Severe Osimertinib-Induced Hepatotoxicity. J. Thorac. Oncol. 12 (5), e61–e63. 10.1016/j.jtho.2017.01.026 28291724

[B25] Zen Y. Yeh M. M. (2018). Hepatotoxicity of Immune Checkpoint Inhibitors: a Histology Study of Seven Cases in Comparison with Autoimmune Hepatitis and Idiosyncratic Drug-Induced Liver Injury. Mod. Pathol. 31, 965–973. 10.1038/s41379-018-0013-y 29403081

